# Effect of 25-hydroxyvitamin D on *Helicobacter pylori* eradication in patients with type 2 diabetes

**DOI:** 10.1007/s00508-018-1416-y

**Published:** 2018-12-12

**Authors:** Bin Huang, Shengju Yan, Chao Chen, Shandong Ye

**Affiliations:** Department of Endocrinology, Anhui Provincial Hospital, 2300000 Hefei, Anhui Province China

**Keywords:** 25-hydroxyvitamin D, Helicobacter pylori eradication, Type 2 diabetes, Lipid profile, Vitamin D deficiency

## Abstract

**Background:**

Various studies have reported a lower *Helicobacter pylori* eradication rate and a more frequent reinfection rate in type 2 diabetes mellitus (T2DM). Vitamin D has anti-inflammatory and immunoregulatory activity and the role of the vitamin D receptor (VDR) in the antimicrobial activity against *H. pylori* has been reported. When it comes to the risk factors of *H. pylori* eradication, the function of vitamin D is not always taken into account. The aim of this study was to assess the role of 25-hydroxyvitamin D in *H. pylori* eradication in T2DM.

**Methods:**

In this retrospective study data from 160 patients with T2DM who underwent eradication therapy for *H. pylori* in Anhui Provincial Hospital between July 2015 and September 2017 were analyzed. According to eradication status, patients were divided into two groups, the successful eradication group (*n* = 124) and the eradication failure group (*n* = 36). The following information was obtained from participants’ records before eradication treatment: age, sex, body mass index (BMI), duration of T2DM, prescription of medication use, smoking and drinking history. All patients were tested for glycated hemoglobin (HbA1c), total cholesterol (TC), high-density lipoprotein cholesterol (HDL-C), low-density lipoprotein cholesterol (LDL‑C), triglyceride (TG) and 25-hydroxyvitamin D (25-OHD) at baseline.

**Results:**

The *H. pylori* was eradicated in 124 (77.5%) patients, while in 36 (22.5%) patients the treatment was unsuccessful. The eradication failure group had a lower mean vitamin D concentration than the group with successful eradication (15.09 ± 7.72 ng/ml vs. 19.87 ± 6.35 ng/ml, *p* = 0.004). The estimated odds ratio (OR) for eradication failure in individuals with serum vitamin D deficiency (<20 ng/ml) compared to those with sufficient vitamin D levels (>30 ng/ml) were 1.489 (95% confidence interval, CI: 1.046–2.121, *P* = 0.027), Individuals with long duration of diabetes (≥10 years) had odds of eradication failure of 1.467 (95% CI: 1.017–2.114, *P* = 0.040) compared to subjects with short duration of diabetes (<10 years).

**Conclusions:**

Lower 25-OHD was not only associated with *H. pylori* eradication failure but was also related to dyslipidemia in T2DM patients. Increasing serum 25-OHD to appropriate levels by activated vitamin D use may improve the eradication rate.

## Introduction

*Helicobacter pylori* is a gram-negative, spiral-shaped, microaerophilic bacterium that plays a major pathogenic role in gastric diseases, resulting in chronic inflammation and immune responses [1]. The inflammatory reactions, which involve many cytokines, including inflammatory cytokines and adipokines, were found to be associated with insulin resistance (IR) and metabolic syndrome (MS) [2]. Moreover, a recent meta-analysis showed that *H. pylori* infections are 1.3-fold more prevalent in persons with diabetes than in those without diabetes and diabetics with *H. pylori* infection had a higher incidence of neuropathy [3]. Various studies have reported a lower eradication rate and a more frequent reinfection prevalence in type 2 diabetes (T2DM) patients [4]. Serum 25-hydroxyvitamin D (25-OHD) is the most proven means to evaluate an individual’s vitamin D status. Vitamin D is in the spotlight as numerous in vitro and animal studies have indicated that vitamin D has anti-inflammatory and immunoregulatory activity, it can play a role in decreasing the risk of various chronic illnesses, including common cancers, autoimmune diseases, infectious diseases, and cardiovascular diseases [5]. The role of the vitamin D receptor (VDR) in the antimicrobial activity against *H. pylori* has been reported [6]. Guo et al. found that the expression of VDR in the gastric epithelium is up-regulated in the case of *H. pylori* infections [7]. This study demonstrated that vitamin D may play an important role in gastric mucosa homeostasis and host protection from *H. pylori* infection. When it comes to the risk factors for *H. pylori* eradication, the function of vitamin D is not always taken into account. The aim of this study was to assess the role of 25-OHD in *H. pylori* eradication in T2DM.

## Subjects and methods

### Subjects

In this retrospective study data from 160 patients with T2DM who underwent eradication therapy for *H. pylori* in Anhui Provincial Hospital between July 2015 and September 2017 were analyzed. The exclusion criteria were as follows: patients with severe osteoporosis, severe hepatic or renal dysfunction, a history of gastric surgery, diagnosis of malignancy, pregnancy and patients with a history of previous *H. pylori* eradication treatment. Patients using antibiotics or proton pump inhibitors within 1 month prior to the study were also excluded.

### Methods

At first, the subjects were diagnosed with *H. pylori* infection by the urea breath test. The eradication therapy for *H. pylori* was performed using oral administration of amoxicillin 2000 mg/day, clarithromycin 1000 mg/day, esomeprazole 40 mg/day with bismuth potassium citrate 440 mg/day for 14 days. The urea breath test was performed in patients after 4 weeks of treatment. According to eradication status, patients were divided into two groups, as the successful eradication group (*n* = 124) and the eradication failure group (*n* = 36). The eradication failure group patients were referred to the gastroenterology outpatient department for appropriate diagnosis and eradication of *H. pylori*. The following information was obtained from participants’ records before eradication treatment: age, sex, body mass index (BMI), duration of T2DM, prescription of medication use, smoking and drinking alcohol history. All patients were tested for glycated hemoglobin (HbA1c), total cholesterol (TC), high (HDL-C) and low-density lipoprotein cholesterol (LDL-C), triglyceride (TG) and 25-OHD at baseline.

### Statistical analysis

For continuous variables, values are expressed as mean ± standard deviations (SD). Categorical data were analyzed with the χ^2^-test or Fisher’s exact test, as appropriate. All statistical tests were 2‑tailed. A *P*-value of <0.05 was considered to indicate a statistically significant difference. The statistical analysis was performed with SPSS version 22.0 (SPSS Inc., Chicago, IL, USA). Differences between the two groups were evaluated by using a Student’s t‑test for continuous measures and χ^2^-test for categorical measures. Multivariate logistic regression analysis was done to identify independent factors affecting the risk of *H. pylori* eradication failure in patients with T2DM. The results of this analysis were expressed as odds ratios (OR) and 95% confidence intervals (CI). Additionally, patients were divided into three groups according to the concentration of serum 25-OHD. The respective subgroups were compared by using one-way ANOVA tests. Post-hoc tests in the one-way ANOVA test.

## Results

Mean age of the T2DM patients was 55.42 ± 11.19 years and 84 (52.5%) of the patients were women. *Helicobacter pylori* was eradicated in 124 (77.5%) patients, while in 36 (22.5%) patients the treatment was unsuccessful. There were no significant differences between the successful eradication and eradication failure groups regarding sex, age, BMI and smoking habits (*p* = 0.677, *p* = 0.328, *p* = 0.509 and *p* = 0.077, respectively). The duration of diabetes (14.58 ± 5.63 years vs. 9.67 ± 3.71 years) and rates of drinking (44.4% vs. 22.6%) were significantly higher in patients with eradication failure (*p* = 0.001 and *p* = 0.01, respectively). The eradication failure group had a lower mean vitamin D concentration than the successful eradication group (15.09 ± 7.72 ng/ml vs. 19.87 ± 6.35 ng/ml, *p* = 0.004). There were no statistically significant differences in vitamin D status distribution, Hba1c, homeostasis model assessment of insulin resistance (HOMA-IR), TC, TG, LDL‑C and HDL‑C. The demographic and clinical data of the patients are presented in Table 1.

**Table 1:** Demographic and clinical data of patients

Variable	All patients	Eradication failure	Eradication successful	*P*-value
Clinical parameters				
Number (male/female)	160 (76/84)	36 (16/20)	124 (60/64)	0.677
Age (years)	55.42 ± 11.19	57.18 ± 12.13	53.58 ± 10.75	0.328
BMI (kg/m^2^)	25.21 ± 3.67	26.46 ± 3.64	25.76 ± 3.89	0.509
Duration of diabetes (years)	11.76 ± 4.59	14.58 ± 5.63	9.67 ± 3.71*	0.001
Drinking alcohol (%)	44 (27.5)	16 (44.4)	28 (22.6)*	0.010
Smoking (%)	36 (22.5)	12 (33.3)	24 (19.3)	0.077
Laboratory parameters				
Hba1c (%)	7.72 ± 2.41	8.14 ± 2.73	7.67 ± 2.22	0.142
HOMA-IR index	4.77 (1.25, 4.79)	4.91 (1.32, 5.24)	4.64 (0.95, 4.18)	0.207
TG, mg/dl	157.48 ± 61.26	162.93 ± 67.32	150.33 ± 59.08	0.345
TC, mg/dl	182.65 ± 70.18	203.85 ± 88.03	170.29 ± 67.42	0.096
LDL-C, mg/dl	128.39 ± 39.41	147.90 ± 47.81	111.25 ± 30.34	0.272
HDL-C, mg/dl	33.59 ± 18.84	30.55 ± 17.38	36.82 ± 19.04	0.520
25-OHD total, ng/ml	17.02 ± 7.16	15.09 ± 7.72	19.87 ± 6.35*	0.004
<20 ng/ml (%)	87 (54.3)	25 (69.5)	62 (50.0)	0.057
20–30 ng/ml (%)	42 (26.3)	8 (22.2)	34 (27.4)	0.668
>30 ng/ml (%)	31 (19.4)	3 (8.3)	28 (22.6)	0.060
Medicine				
Mealtime insulin (%)	53 (33.1)	13 (36.1)	40 (32.3)	0.690
Sulfonylureas (%)	51 (31.9)	12 (33.3)	39 (28.42)	0.841
DPP-4 inhibitor (%)	40 (25)	9 (25.9)	31 (25.0)	1.000
Metformin (%)	62 (38.8)	15 (40.7)	47 (37.9)	0.701
Alpha-glucosidase inhibitor (%)	14 (8.8)	3 (8.3)	11 (8.9)	1.000
Aspirin (%)	88 (55.0)	21 (58.3)	67 (54.0)	0.706
Statins (%)	106 (66.3)	22 (61.1)	84 (67.7)	0.549
Activated vitamin D (%)	68 (42.5)	7 (19.4)	61 (49.2)*	0.001
Calcium (%)	87 (54.4)	15 (41.7)	72 (58.64)	0.090

*p < 0.05 vs. eradication failure group

*BMI* body mass index, *HOMA-IR* homeostasis model assessment of insulin resistance, *TC* total cholesterol, *HDL‑C* high-density lipoprotein cholesterol, *LDL‑C* low-density lipoprotein cholesterol, *TG* triglyceride, *25-OHD* 25-hydroxyvitamin D, *Hba1c* glycated hemoglobin

In addition, multivariate analysis was performed by stepwise discriminant analysis to identify variables independently associated with the risk of *H. pylori* eradication failure in T2DM patients. The multivariate analysis controlled for all factors with significant associations emerging from the univariate analysis. The estimated OR for eradication failure in individuals with serum vitamin D deficiency (<20 ng/ml) compared to those with sufficient vitamin D levels (>30 ng/ml) were 1.489 (95% CI: 1.046–2.121, *P* = 0.027), Individuals with a long duration of diabetes (≥10 years) had the OR of eradication failure of 1.467 (95% CI: 1.017–2.114, *P* = 0.040) compared to subjects with short duration of diabetes (<10 years). The failure risk was not significantly increased when patients who were drinking alcohol (95% CI: 0.769–1.637, *P* = 0.212; Table 2).

**Table 2:** Mutually adjusted odds ratios for *Helicobacter pylori* eradication

Factor	B	*P*-value	Exp(B)	95%CI	
Vitamin D (<20 ng/ml)	0.398	0.027	1.489	1.046	2.121
Vitamin D (20–30 ng/ml)	0.347	0.133	1.415	0.899	2.228
Duration of diabetes (≥10 years)	0.383	0.040	1.467	1.017	2.114
Drinking alcohol	0.259	0.212	1.203	0.769	1.637

*B*, *Exp(B)*, *CI* confidence interval

**Fig. 1:**
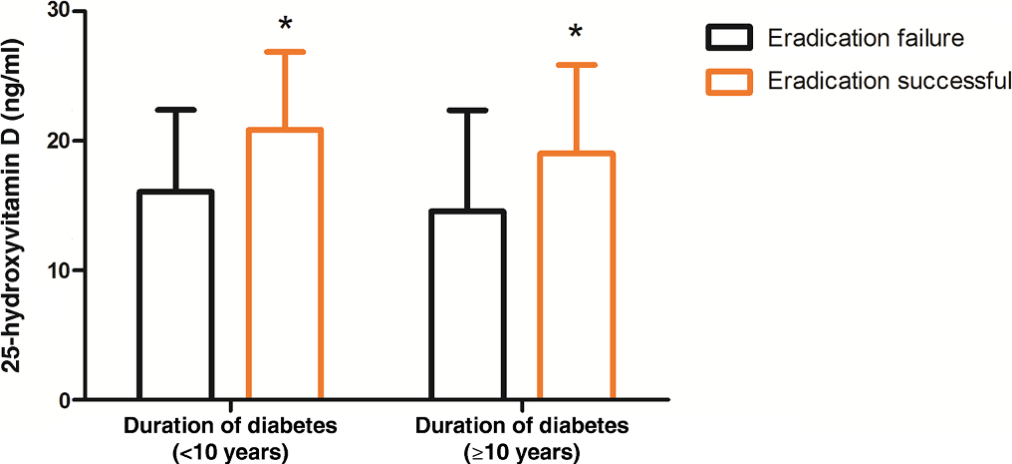
The serum concentration of 25-hydroxyvitamin D by duration of diabetes stratification in eradication failure or success patients (*P < 0.05 successful eradication vs. eradication failure group)

As both vitamin D level and duration of diabetes were correlated with eradication outcome, a further analysis with duration of diabetes stratification was performed to compare serum vitamin D levels. No significant intergroup difference in serum vitamin D level was found between individuals with *H pylori* eradication successful and failure (Fig. 1).

Baseline levels of 25-OHD were classified as deficient (<20 ng/ml), insufficient (20–30 ng/ml), and normal (>30 ng/l). A further subgroup analysis was performed to compare the traits of the patients in these three groups. The eradication rate was significant lower in vitamin D deficiency patients compared to sufficient vitamin D patients (71.3% vs. 90.3%, *p* = 0.032). Individuals with vitamin D deficiency had a higher TC, LDL-C and lower HDL-C level than those with vitamin D levels > 30 ng/l (*p* = 0.018, *p* = 0.047 and *p* = 0.010, respectively). There were no significant differences in the remaining traits, including TG, Hba1c and HOMA-IR between these 3 groups (*p* > 0.05; Table 3).

**Table 3:** Traits of the patients in different 25-OHD levels

	Vitamin D (<20 ng/ml)	Vitamin D (20–30 ng/ml)	Vitamin D (>30 ng/ml)
Hba1c, %	8.05 ± 3.12	7.68 ± 2.29	7.71 ± 3.66
HOMA-IR index	5.14 (2.03, 5.44)	4.65 (1.38, 4.92)	4.39 (1.09, 4.82)
TG, mg/dl	169.44 ± 72.03	160.26 ± 68.32	146.93 ± 81.24
TC, mg/dl	240.75 ± 77.80*	181.28 ± 63.90	163 ± 58.24
LDL-C, mg/dl	157.29 ± 31.97*	124.43 ± 45.02	105.89 ± 54.02
HDL-C, mg/dl	22.89 ± 10.82*	34.87 ± 21.15	41.27 ± 20.09
HP eradication, %	71.3*	81.0	90.3
Activated vitamin D	8	36	24

*p < 0.05 vs. normal group (25-hydroxyvitamin D > 30 ng/l)

The patients were interviewed about their medication use on admission. These medications included antidiabetics, aspirin, statins, activated vitamin D, calcium. It was assumed that a patient was actively taking these drugs if there was a regular use during the last 90 days. Among the successful group, activated vitamin D usage was 61/124 (49.2%), whereas in the failure group activated vitamin D usage was 7/36 (19.4%) cases (χ^2^ = 10.104, *p* = 0.001). There was no statistical difference in residual drug use between the two groups (Table 1). According to activated vitamin D treatment or not, patients were divided into two groups. In this research the activated vitamin D treatment group had a higher *H pylori* eradication rate compared to the group without treatment (85.35% vs. 71.7%, *p* = 0.025); however, in the logistic regression analysis with adjustments for serum 25-OHD, individuals with vitamin D usage had the odds of eradication failure of 0.791 (95% CI: 0.429–1.270, *P* = 0.142) compared to subjects without vitamin D therapy.

## Discussion

Recently, it has been stated that infections with *H pylori* may have a pathogenic role in diabetes mellitus. The *H pylori* might condition the pathophysiology of autoimmune response and insulin resistance syndrome by pathologic consequences through chronic inflammation outside the stomach, by which the bacterium affects glycemic control in diabetic patients. In another aspect, gastrointestinal conditions related to *H pylori* infections could delay gastric emptying, consequently favoring poor glucose control [8]. Studies have reported that HP infections cause microvascular damage and trigger premature development of atherosclerosis in diabetic patients [9]. Gulcelik et al. reported that there is an important relationship between *H pylori* -positivity and neuropathy [10]. A favorable effect of *H. pylori* eradication on insulin resistance was also documented in some studies [11]. Yanik et al. found that *H. Pylori* eradication has a favorable effect on reducing microalbuminuria in diabetic patients [12]. Unfortunately, various studies have reported a lower *H. Pylori* eradication rate and a more frequent reinfection prevalence in diabetic patients [13]. The issue of outcomes of attempted *H pylori* eradication in diabetic patients has long been neglected in the medical literature and does not feature in the relevant guidelines. Studies have shown that antibiotic resistance, virulence factors, host-related genetic disorders and other factors, such as inadequate drug adherence, smoking habits, or alcohol consumption are associated with *H. pylori* eradication failure [14, 15]. In this study patients were treated with a bismuth-containing quadruple eradication therapy, which is recommended as a first-line treatment in regions where clarithromycin resistance is prevalent. The studies have shown that bismuth-containing quadruple therapy leads to eradication rates of 77.5% in T2DM patients. As the low eradication rate, there is a imperative to elucidate factors that may interfere with the H. pylori eradication in T2DM.

In a retrospective study, Yildirim et al. suggested that 25-OHD deficiency may be considered a risk factor related to *H. pylori* eradication failure [16]. Patients with diabetes have been demonstrated to have low vitamin D levels [17]. In this research, the concentration of vitamin D and vitamin D status distribution were significant differences between 160 *H. pylori* infection patients with T2DM and 160 *H. pylori* infection age and BMI-matched patients without T2DM. Further analysis noted that a higher concentration of vitamin D was associated with higher *H. pylori* eradication rates and demonstrated that vitamin D may also play an important role in the process of *H. pylori* eradication in T2DM patients. Host immunity plays a major role against an infectious disease such as *H. pylori* infection. Vitamin D, beyond its well-known role in bone formation, also has an immunomodulator role in targeting various immune cells [16]. Vitamin D deficiency may increase the incidence of immune system disorders and may be a risk factor for the progression of an infectious disease. Logistic analysis yielded an OR of 1.489 for eradication failure in patients with vitamin D deficiency (<20 ng/ml) compared to subjects with normal vitamin D levels (>30 ng/ml). A potential pathogenic mechanism explaining the observed association between vitamin D status and eradication rates is impairment of the vitamin D signal, which may result in inadequate immune response [5]. There are limited data demonstrating the relationship between vitamin D and *H. pylori* infection. One in vitro study showed the selective antibacterial effect of vitamin D3 decomposition product (VDP1) against *H. pylori* [18]. Vitamin D is also known to regulate the expression of the antimicrobial peptide cathelicidin, which kills the bacteria [19]. Another antimicrobial peptide β‑defensin, which is secreted in the gastric mucosa after infection by *H. pylori*, constitutes immune defence against this bacterial pathogen at the mucosal surface [20]. In a vitamin D-deficient state, the infected macrophage is unable to produce sufficient 1,25-OHD to upregulate the production of cathelicidin and β‑defensin, thus rendering them unable to kill the *H. pylori* strains [21]. it was also found that short diabetes duration was associated with higher *H. pylori* eradication rates. The OR was 1.467 for eradication failure in patients with diabetes duration more than 10 years. The subgroup analysis with duration of diabetes stratification indicated that the negative effect of long diabetes duration was independent of vitamin D levels.

Adult studies have reported associations of low 25-OHD with dyslipidemia and cardiovascular disease [22]. Vitamin D deficiency was significantly associated with increase in atherogenic lipids and markers of early cardiovascular disease [23]. These findings suggest that vitamin D deficiency may have negative effects on lipid parameters. In this study, vitamin D deficiency was found to be significantly associated with an increase in atherogenic lipids. Studies found that 1,25-dihydroxycholecalciferol activates CYP3A4, an enzyme of the cytochrome P450 system, which metabolizes statins to the main metabolites [24, 25]. The lack of vitamin D may cause the statins to work less effectively and this may also explain the uncorrelation between TG and vitamin D.

Our results suggested that higher 25-OHD may be beneficial to *H. pylori* eradication. The use of activated vitamin D in T2DM patients is very common. Obviously, it can improve serum 25-OHD so that the eradication rate is increased. Whether the drug protects gastric mucosa and antimicrobial directly is not clear. Studies have found that 1,25(OH)2D binds to VDR, activates VDR signalling, and induces a series of antimicrobial responses [7]. Hosoda et al. demonstrated that the vitamin D (3) decomposition product VDP1 exerts an antibacterial action against *H. pylori* [18]. In our research, logistic regression analysis with adjustments for serum 25-OHD, the improvement of eradication rate by activated vitamin D therapy disappeared. This result shows that the anti-* H. pylori* effect of activated vitamin D is not acting on gastrointestinal tract directly.

In conclusion, the results suggested that lower 25-OHD was not only associated with *H. pylori* eradication failure but also related to dyslipidemia in T2DM patients. Increasing serum 25-OHD to appropriate levels by activated vitamin D use may improve eradication rate. Given the importance of this subject, more studies are warranted to further understand the effect of supplementing vitamin D in the *H. pylori* eradication treatment.
